# Sudden-Onset Disaster Mass-Casualty Incident Response: A Modified Delphi Study on Triage, Prehospital Life Support, and Processes

**DOI:** 10.1017/S1049023X23006337

**Published:** 2023-10

**Authors:** Joe Cuthbertson, Eric Weinstein, Jeffrey Michael Franc, Peter Jones, Hamdi Lamine, Sabina Magalini, Daniele Gui, Kristina Lennquist, Federica Marzi, Alessandro Borrello, Pietro Fransvea, Andrea Fidanzio, Carlos Yanez Benítez, Gerhard Achaz, Bob Dobson, Nabeela Malik, Michael Neeki, Ronald Pirrallo, Rafael Castro Delgado, Giacomo Strapazzon, Marcelo Farah Dell’Aringa, Hermann Brugger, Chaim Rafalowsky, Marcello Marzoli, Giovanni Fresu, Knut Magne Kolstadbraaten, Stenn Lennquist, Jonathan Tilsed, Ilene Claudius, Piyapan Cheeranont, Rachel Callcut, Miklosh Bala, Anthony Kerbage, Luis Vale, Norman Philipp Hecker, Roberto Faccincani, Luca Ragazzoni, Marta Caviglia

**Affiliations:** 1. CRIMEDIM – Center for Research and Training in Disaster Medicine, Humanitarian Aid, and Global Health, Università del Piemonte Orientale, Novara, Italy; 2. Monash University Disaster Resilience Initiative, Monash University, Clayton VIC Australia; 3. Department of Emergency Medicine, University of Alberta, Edmonton, AB, Canada; 4. Assistance Publique – Hópitaux de Paris (APHP), SAMU de Paris Hôpital Necker, Paris, France; 5. Department for Sustainable Development and Ecological Transition, Università del Piemonte Orientale, Vercelli, Italy; 6. Department of Surgery, Catholic University of the Sacred Heart, Policlinico Gemelli, Rome, Italy; 7. Department of Neurosciences, Catholic University of the Sacred Heart, Policlinico Gemelli, Rome, Italy; 8. Department of Surgery, San Jorge University Hospital, Huesca, Spain; 9. London Ambulance Service NHS Trust, London, London, United Kingdom; 10. University Hospitals Birmingham NHS Trust, Edgbaston, Birmingham, United Kingdom; 11. Clinical Professor of Emergency Medicine, Arrowhead Regional Medical Center, Colton, California USA; Professor of Medical Education, California University of Science and Medicine, Colton, California USA; 12. Department of Emergency Medicine, Prisma Health University of South Carolina School of Medicine Greenville, Greenville, South Carolina USA; 13. Health Service of the Principality of Asturias (SAMU-Asturias), Health Research Institute of the Principality of Asturias (Team Leader of the Research Group on Prehospital Care and Disasters, GIAPREDE), Oviedo, Spain; 14. Department of Medicine, Oviedo University, Oviedo, Spain; 15. Institute of Mountain Emergency Medicine, Eurac Research, Bolzano, Italy; University of Padova, Padova, Italy; International Commission for Mountain Emergency Medicine, Zurich, Switzerland; 16. Institute of Mountain Emergency Medicine, Eurac Research, Bolzano, Italy; Medical University Innsbruck, Innsbruck, Austria; International Commission of Mountain Emergency Medicine-ICAR MedCom, Zurich, Switzerland; 17. Department of General Surgery, Hadassah Medical Center and Faculty of Medicine, Hebrew University of Jerusalem, Israel; 18. Department of Fire Service, Public Rescue and Civil Defence, Ministero dell’Interno, Rome, Italy; 19. Department of Traumatology, Oslo University Hospital – Ullevaal, Oslo, Norway; 20. Department of Emergency Medicine, Harbor-UCLA, Torrence, California USA; 21. Faculty of Medicine, Praboromarajchanok Institute, Ministry of Public Health, Nonthaburi, Thailand; 22. University of California Davis Department of Surgery, Sacramento, California USA; 23. Department of Internal Medicine, Hôtel-Dieu de France hospital, Beirut, Lebanon; 24. ESTES—European Society for Trauma and Emergency Surgery, Disaster and Military Surgery Section, Milan, Italy; 25. Department of Translational Medicine, Università del Piemonte Orientale, Novara, Italy

**Keywords:** disaster, mass casualty, prehospital, trauma

## Abstract

The application and provision of prehospital care in disasters and mass-casualty incident response in Europe is currently being explored for opportunities to improve practice. The objective of this translational science study was to align common principles of approach and action and to identify how technology can assist and enhance response. To achieve this objective, the application of a modified Delphi methodology study based on statements derived from key findings of a scoping review was undertaken. This resulted in 18 triage, eight life support and damage control interventions, and 23 process consensus statements. These findings will be utilized in the development of evidence-based prehospital mass-casualty incident response tools and guidelines.

## Introduction

Increasing frequency and magnitude of disasters bring to light the constantly emerging risks and the planetary health-related consequences of their impact.^1,2^ Consequently, a more integrated approach to prevent and quickly respond to the threat of hazards becoming sudden-onset disasters and mass-casualty incidents is urgently needed.^3,4^ Within this goal, the Horizon 2020 Novel Integrated Toolkit for Enhanced Prehospital Life Support and Triage in Challenging and Large Emergencies (NIGHTINGALE) project has been established to support preparedness of first responders during sudden-onset disasters and mass-casualty incidents through the description of evidence-based guidelines for mass-casualty incident triage, prehospital life support and damage control interventions, and prehospital processes together with the creation of a series technological tools that will enhance the first responders capabilities.^5^ The need to improve the preparedness and capability of first responders to plan for and respond to these events is consistent with published findings identifying that triage and organization in mass-casualty incidents is a prehospital research priority.^6^ This goal is consistent with the Sendai Framework for Disaster Risk Reduction, which identifies the need to improve health system resilience and develop local capacity at all health levels in reducing and addressing disaster risk.^7^ The nature of sudden-onset disasters that produce mass-casualty incidents creates challenges in conducting research, such as randomized control trials and conventional prospective studies, due to the unpredictable and uncontrolled nature of the events that lead to more exploratory research methodologies being applied in many cases.^8,9^ To overcome this, a modified Delphi study has been conducted to answer the research question: What are the common denominators in the provision of mass-casualty incident triage, prehospital life support and damage control, and prehospital processes to enhance operational capacities during the prehospital management of mass-casualty incidents?^10^

A Preferred Reporting Items for Systematic Reviews and Meta-Analyses Extension for Scoping Reviews (PRISMA-ScR) scoping review was performed^11^ that extracted and synthesized the evidence base of mass-casualty incident triage, prehospital life support and damage control, and prehospital processes. The findings of this scoping review informed the development of statements utilized in this modified Delphi method study. This expert-based method is widely used to reach consensus and explore assumptions and alternatives.^12–14^ This study aimed to produce mass-casualty incident triage, prehospital life support and damage control, and prehospital process consensus statements that will be incorporated in the third stage development of evidence-based prehospital mass-casualty incident response tools and guidelines.

## Methods

**Figure 1.**
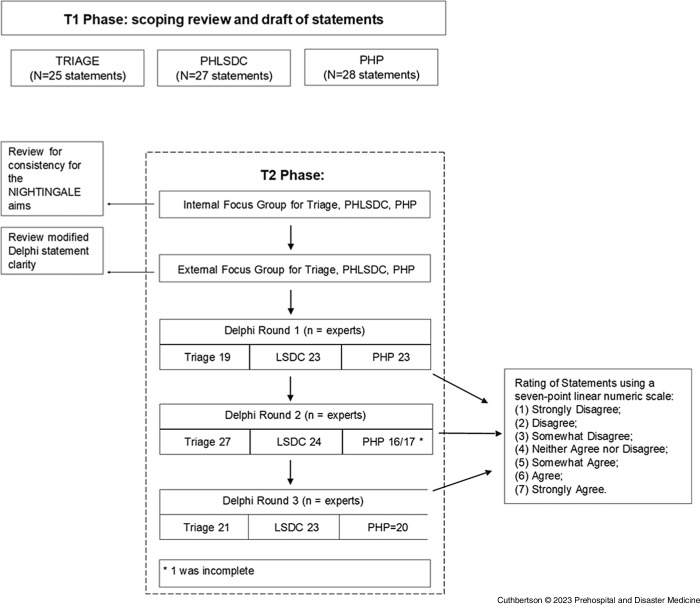
Modified Delphi Flowchart. Abbreviations: PHLSDC, prehospital life support and damage control interventions; PHP, prehospital processes; LSDC, life support and damage control interventions; NIGHTINGALE, Novel Integrated Toolkit for Enhanced Prehospital Life Support and Triage in Challenging and Large Emergencies.

The modified Delphi technique used in this study differs from the standard Delphi approach of using an open questionnaire to retrieve expert data from which these experts offer to create statements (Figure 1). Once statements are created, these experts provide their opinions to achieve group consensus in subsequent Delphi stages.^15^ The modification of this study applied the outcomes of a previously conducted PRISMA-ScR scoping review to capture data related to the research topic in a robust, valid manner.^16^

Data retrieved has been analyzed and synthesized into three initial sets of statements, brought to the attention of internal focus groups (IFGs) and external focus groups (EFGs) to produce the final Delphi statements (Figure 1).

Three IFGs for mass-casualty incident triage, prehospital life support and damage control, and prehospital processes were conducted in parallel in January and February 2022. Participants comprised expert practitioners and researchers in mass-casualty incident response. Experts were engaged in three parallel one-hour video conferences and then exchanged drafts via email to review the three sets of draft statements produced from the scoping review, to render them clear, concise, and consistent with the objectives of NIGHTINGALE.

To reduce risk of bias from the statement creation process, three EFGs were conducted. Participants included international experts not engaged in the NIGHTINGALE project, comprising practitioners and researchers in the field of mass-casualty incident triage, prehospital life support and damage control, and prehospital processes, who were identified as authors of relevant references discovered during the scoping review, or members of scientific societies, namely the European Society for Trauma and Emergency Surgery (ESTES; Vienna, Austria), the World Association for Disaster and Emergency Medicine (WADEM; Madison, Wisconsin USA), and the National Association of Emergency Medical Services Physicians (NAEMSP; Atlanta, Georgia USA). Experts participated in three parallel one-hour video conferences and then exchanged drafts via email, intending to ensure that the three sets of statements met the specifics of the Delphi format, that statements are preferred over questions, and that one statement discusses one fact.

### Delphi Rounds

The three expert round modified Delphi were conducted from March 14 through April 11, 2022 using the Stat59 platform (STAT59 Services Ltd; Edmonton, Alberta, Canada). Recruited experts included operational first responders, academic researchers identified among the authors of included scoping review references, alumni of the European Master of Disaster Medicine (EMDM), and members of the professional scientific societies that were not focus group participants from ESTES, WADEM, and NAEMSP as experts in the field of either mass-casualty incident triage, prehospital life support and damage control, or prehospital processes. Experts that did not meet these criteria were excluded.

In the present research, the rationale underlying the selection of experts was based on their geographic distribution and the heterogeneity of the overall group of experts in terms of domains of expertise to establish geographic coverage and a balanced distribution of expertise related to disaster management practices considered.

Following the methodology of Weinstein, et al,^17^ experts who agreed received a formal explanation of the modified Delphi, and the first modified Delphi round questionnaire with 25 triage, 27 prehospital life support and damage control, and 28 prehospital process statements with instruction to rank each statement on a seven-point linear numeric scale with one = “Strongly Disagree” to seven = “Strongly Agree” and four demographic questions. Consensus among experts was defined as a standard deviation (SD) ≤1.0.

Statements that attained consensus after this first expert round were included in the final report, while those that were not in agreement but reached consensus were removed from further consideration. Statements not reaching consensus advanced to the second expert round. For this second expert round (and subsequent rounds if required), the mean response of the experts for the remaining statements and their own response for each of them were displayed. The experts were asked to reconsider their seven-point linear numeric scale. The final report lists all statements reaching consensus.

The McLeod Health Institutional Review Board Office (Florence, South Carolina USA) has determined that this study does meet the exemption criteria found at 45 CFR 46.104(d)(2).^18^

### Data Analysis

Descriptive statistics of the mean and SD were calculated. The response rate was calculated as the percentage of experts who responded in each round (Table 5).

## Results

Sixty-two (62) international experts were recruited to participate in the modified Delphi study. Recruitment demographic characteristics of the recruited experts are presented in Table 1. The outcomes of the three modified Delphi expert rounds are illustrated in Table 2.

**Table 1.** Demographic Characteristics of Delphi Experts

	Triage, n (%) 18 experts	PHLSDC, n (%) 22 experts	PHP, n (%) 22 experts
Years of Expertise in the Field:			
Less < than 5	9 (50.0%)	2 (9.1%)	1 (4.5%)
Less < than 10	3 (16.7%)	6 (27.3%)	4 (18.2%)
Less < than 15	0 (0.0%)	3 (13.6%)	5 (22.7%)
Less < than 20	5 (27.7%)	6 (27.3%)	1 (4.5%)
Greater > than 20	1 (5.5%)	2 (9.1%)	10 (45.5%)
N/A	0 (0.0%)	3 (13.6%)	1 (4.5%)
Location of Mass-Casualty Practice (World Bank Regions)			
East Asia and Pacific	2 (11.1%)	2 (9.1%)	–
Europe and Central Asia	11 (61.1%)	13 (59.1%)	14 (63.6%)
Middle East and North Africa	1 (5.6%)	3 (13.7%)	5 (22.7%)
North America	3 (16.7%)	4 (18.2%)	2 (9.1%)
Sub-Saharan Africa	1 (5.6%)	–	1 (4.5%)
Primary Employment			
Governmental Organization	7 (38.9%)	11 (50.0%)	9 (40.9%)
Nongovernmental Organization	3 (16.7%)	2 (9.1%)	2 (9.1%)
Private Sector	2 (11.1%)	4 (18.2%)	2 (9.1%)
University	4 (22.2%)	4 (18.2%)	7 (31.8%)
Other	2 (11.1%)	1 (4.5%)	2 (9.1%)
Current Profession (Multiple Choice Allowed)			
Administration and Support	3	4	4
EMT/Paramedic	2	–	3
Education/Training	9	15	2
Fire Fighter	–	–	2
Nurse	–	–	2
Physician	16	21	16
Public Safety	–	1	2
Research	7	8	14
Response/Field Operations	7	9	10
Simulation Coder/Designer/Creator	1	5	3
Other	3	1	2

Abbreviation: PHLSDC, prehospital life support and damage control interventions; PHP, prehospital processes; EMT, emergency medical technician.

**Table 2.** Modified Delphi Statement Consensus Outcomes

	Modified Delphi Outcomes					
Theme	Initial	Round 1	Round 2	Round 3	Attained	Not Attained
Triage	25	10	4	4	18	7
Prehospital Life Support and Damage Control	27	2	4	2	8	19
Prehospital Processes	28	11	11	1	23	5
Total	80	23	19	7	49	31

The consensus outcomes of this study were 18 mass-casualty incident triage, eight prehospital life support and damage control, and 23 prehospital process statements, as shown in Table 3.

**Table 3.** Triage, Prehospital Life Support and Damage Control, and Prehospital Process Statements that Achieved Consensus

Theme	Statements	Mean Score	Standard Deviation
**Triage**	Triage is an on-going and repetitive process throughout the continuum from the initial assessment through deﬁnitive care.	6.6	0.7
	Each triage system should allow for dynamic triage decisions based on changes in available treatment and transportation resources.	6.4	1.0
	Each triage system should be inclusive of all populations.	6.3	1.0
	Each triage system should be simple, easy to remember, amenable to quick memory aids, and just-in-time training for trained ﬁrst responders.	6.7	0.7
	Each triage system should be practical for use in an austere environment.	6.5	0.7
	Each triage system should require that the assigned triage category for each patient be visibly identiﬁable and/or by patients being sent to a speciﬁc assigned location as a group of similar triaged patients.	6.3	0.9
	It should be possible to perform the initial assessment without diagnostic equipment.	6.5	0.8
	Patients categorized or considered as expectant should be provided with treatment and/or transport as resources become available.	6.6	0.7
	Efﬁcient use of transport assets may include mixing categories of patients and using alternate forms of transport.	6.2	0.5
	Use of ultrasound may be incorporated in the continuum of prehospital care.	6.2	1.0
	Each jurisdiction should require that all ﬁrst response agencies utilize the same triage system for any mass-casualty incident response in that jurisdiction.	6.5	0.9
	Each triage system should allow for dynamic triage decisions based on changes in patient conditions.	6.6	0.7
	Each triage system should be inclusive of all ages.	6.0	0.7
	The ﬁeld trauma score may be used to guide life-saving and damage control interventions.	5.0	0.9
	Each jurisdiction should develop clinical guidelines for priority transportation decisions to match the patient to the appropriate deﬁnitive health care facility.	5.7	0.9
	Each triage system should develop a continuum of repeated assessments of available vital signs.	5.8	0.9
	Each jurisdiction should develop clinical guidelines for priority life support and damage control intervention.	5.4	1.0
	Each ﬁrst response agency should develop protocols for use of monitoring equipment.	5.2	0.9
**Prehospital Life Support and Damage Control**	Pain management should be considered for the injured and when performing interventions.	6.7	0.7
	Each jurisdiction should document life support and damage control intervention in a patient care record.	6.3	0.6
	Each medical ﬁrst response agency should develop crush injury treatment guidelines, education, and training to achieve and maintain competencies.	6.2	1.0
	Each medical ﬁrst response agency should develop clinical guidelines, education, and training to achieve and maintain competencies to utilize intraosseous access to achieve rapid vascular access.	6.4	0.9
	Each ﬁrst response agency should utilize a formal evidence-based framework for post-incident evaluation that deﬁnes and assesses key performance indicators.	6.0	1.0
	Each jurisdiction should create guidelines to utilize spontaneous ﬁrst providers/bystanders.	5.6	0.9
	Each medical ﬁrst response agency should develop permissive hypotension clinical guidelines, education, and training to achieve and maintain competencies.	5.9	1.0
	After life-saving interventions are performed, the continued monitoring of the patient can be assigned to a provider of lesser training (ie, physician to paramedic or Emergency Medical Technician/EMT, paramedic to EMT or ﬁrst responder with medical training).	5.4	1.0
**Prehospital Processes**	Each jurisdiction’s prehospital processes should be inclusive of all populations.	6.4	0.9
	Each jurisdiction should develop contingency plans for casualty collection points (ie, advanced medical posts, ﬁeld hospitals, alternate care sites, repurposing health care facilities) to meet the demand of mass-casualty incident response.	6.6	0.5
	If available, each jurisdiction should apply technology to recognize and locate emergency response vehicles at all times.	6.2	1.0
	Transport information management systems enhance coordination of patient distribution.	6.6	0.8
	Information management systems enhance coordination of resources (ie, staff, stuff, structures).	6.5	0.8
	Each jurisdiction should have contingencies to manage transport disruption caused by a mass-casualty incident (ie, earthquake destroying road/rail).	6.5	0.6
	Each jurisdiction should apply evidence-based key performance indicators to evaluate and improve the mass-casualty incident response.	6.4	0.8
	The mass-casualty incident response plan should be based on the jurisdiction hazard vulnerability and risk analysis.	6.3	1.0
	Each jurisdiction mass-casualty incident response plan should include a structured debrief of the exercise or actual mass-casualty incident by all participating first response agencies, where possible.	6.4	0.8
	Each jurisdiction should ensure mass-casualty incident response plan education, training, and competencies are consistent across first response agencies.	6.6	0.6
	Assessment, observation, and monitoring technology and devices that have capacity for storing and transmitting data enhance mass-casualty incident response.	5.9	0.8
	Each jurisdiction should deﬁne mass-casualty incident response terminology utilized by all ﬁrst response agencies in this jurisdiction.	6.6	0.6
	Each jurisdiction’s prehospital processes should be inclusive of all ages.	6.5	0.7
	Each jurisdiction should develop search and rescue guidelines.	6.2	1.0
	Each jurisdiction should develop search and rescue education, training, and competencies.	6.2	1.0
	Each jurisdiction should develop mass-casualty incident Chemical, Biological, Radiological, and Nuclear (CBRN) decontamination education, training, and competencies.	6.2	0.9
	Each jurisdiction should develop communication technology backup for all ﬁrst response agencies in the jurisdiction.	6.6	0.7
	Each jurisdiction should develop a single patient identification method utilized across all first response agencies.	6.3	1.0
	Evaluation of an exercise or actual mass-casualty incident event should be completed by all participating first response agencies.	6.5	0.6
	Each jurisdiction mass-casualty incident plan should be designed to be consistent with the jurisdictional incident management system.	6.8	0.4
	Each jurisdiction mass-casualty incident response plan should be designed to be consistent with the jurisdictional health authority legislation and regulations.	6.4	0.9
	Unmanned aerial vehicle/UAV technology enhances mass-casualty incident response situational awareness.	5.5	0.8
	Unmanned aerial vehicle/UAV technology enhances mass-casualty incident response operations.	5.7	0.9

Abbreviations: EMT, emergency medical technician; CBRN, Chemical, Biological, Radiological, and Nuclear; UAV, unmanned aerial vehicle.

The statements that did not reach consensus outcomes of this study were six triage statements, 17 prehospital life support and damage control statements, and five prehospital processes statements, as shown in Table 4.

**Table 4.** Triage, Prehospital Life Support and Damage Control, and Prehospital Process Statements that Did Not Achieve Consensus with SD >1.0 after Three Delphi Expert Rounds

Theme	Statements	Mean Score
**Triage**	1. Patients should be assigned a triage category, as deﬁned by the ﬁrst response agency, according to their condition after any life-saving interventions.	5.2
	2. Each ﬁrst response agency should develop speciﬁc education, training, and competencies for their speciﬁc jurisdiction triage system.	5.8
	3. Each triage system should be based on the jurisdiction hazard vulnerability and risk analysis.	4.7
	4. The shock index may be used to guide life-saving and damage control interventions.	4.2
	5. The pulse pressure may be used to guide life-saving and damage control interventions.	3.9
	6. The heart rate variability may be used to guide life-saving and damage control interventions.	4.0
**Prehospital Life Support and Damage Control**	1. Each ﬁrst response agency should develop crush injury incident recognition guidelines, education, and training to achieve and maintain competencies.	5.6
	2. Materials for crush injury resuscitation should be included on every medical ﬁrst response vehicle.	5.2
	3. Each ﬁrst response agency should develop life-threatening hemorrhage control guidelines, education, and training to achieve and maintain competencies.	6.6
	4. Each ﬁrst response agency should develop life-threatening hemorrhage ﬁrst provider/bystander training and education programs that interface with the ﬁrst response agency.	5.7
	5. Each ﬁrst response agency should develop CBRNE incident recognition, education, and training to achieve and maintain competencies.	5.7
	6. Each ﬁrst response agency should develop CBRNE incident clinical guidelines, education, and training to achieve and maintain competencies.	5.1
	7. Each ﬁrst response agency should develop an awareness of the need for speciﬁc CBRNE antidotes and to deliver these to the scene.	4.6
	8. When feasible, the medical ﬁrst response agency should develop blood product use clinical guidelines, education, and training to achieve and maintain competencies and to deliver these to the scene.	4.9
	9. Each medical ﬁrst agency should develop tranexamic acid (TXA) clinical guidelines, education, and training to achieve and maintain competencies.	5.8
	10. Each ﬁrst response agency should develop mass hypothermia monitoring and treatment guidelines.	5.5
	11. First responders should only perform interventions within their scope of practice.	6.0
	12. Each ﬁrst response agency should develop smoke inhalation education and training to achieve and maintain competencies.	5.7
	13. Each medical ﬁrst response agency should develop clinical guidelines, education, and training to achieve and maintain competencies to treat the speciﬁc patient with hypotension due to hemorrhage and a declining level of consciousness without clear evidence of a head injury.	6.5
	14. Each medical ﬁrst response agency should develop blunt and penetrating chest trauma education and training to achieve and maintain competencies.	6.3
	15. Deployable technology should employ evidence-based physiologic parameters and undergo clinical evaluation before utilization.	5.8
	16. Each medical ﬁrst response agency should evaluate the use of the motor Glasgow Coma Score (mGCS) in certain clinical scenarios in preference to the total Glasgow Coma Score (tGCS).	4.1
	17. Each jurisdiction should utilize a patient consent process for interventions.	4.3
**Prehospital Processes**	1. Each jurisdiction should develop mass-casualty incident CBRNE decontamination guidelines.	6.0
	2. Each jurisdiction should define futility of care.	5.0
	3. Each jurisdiction should develop community response education and training to active shooter.	5.2
	4. Each jurisdiction should develop first responder plans, education training, and competencies for active shooter events.	5.4
	5. Where possible, a jurisdiction should apply telemedicine technology and processes to support the mass-casualty incident event response.	5.6

Abbreviation: CBRNE, Chemical, Biological, Radiological, Nuclear, and Explosives

**Table 5.** Delphi Expert Response Rate

	Number of Delphi Experts			Response Rate = # of Delphi Experts in this Round/ Initial # of Delphi Experts		
	Triage	Prehospital Life Support and Damage Control	Prehospital Processes	Triage	Prehospital Life Support and Damage Control	Prehospital Processes
**First Round**	19	23	23			
**Second Round**	27	24	16/17^a^	27/19 = 1.42^b^	24/23 = 1.04^b^	16/23 = 0.69; 17/23 = 0.74
**Third Round**	21	23	20	21/19 = 1.10^c^	23/23 = 1^c^	20/23 = 0.87

a This Round 1 expert offered their opinion on 5/17 statements of which five reached group consensus and 12 did not reach consensus.

b More experts participated in the second round.

c Some experts left the third round after participating in the second round.

This study produced nine statements that reached a consensus that referred to components of mass-casualty incident triage systems. Key themes of triage practices that the statements identified included the following:

- A singular triage system should be consistently applied by the agency or agencies and be inclusive of all ages and populations.
- First responders’ initial triage should be simplified and done without the aid of diagnostic equipment, and it should produce a clear indicator of the patient triage category.
- Mass-casualty incident triage is an on-going process clinically guided, and priority categories should be revised with frequent re-assessments guided by patient clinical status after response to life support and damage control interventions, which should change accordingly to the response to these interventions as more resources become available with the goal to achieve priority transportation.

Additionally, six triage statements met consensus with themes of triage application accuracy, first responder agency competency, and protocol to develop clinical triage key performance indicators. There was limited consensus of statements on practice related to prehospital life support and damage control theme. Of the 27 statements, only eight met consensus, of which respondents were in favor of guideline development for pain relief, documentation of care, rapid vascular access utilizing intraosseous access, hypotension management, scope of care, and outcomes measurement. Key areas of treatment guidance that did not meet statement consensus included first responder/first response agency hemorrhage treatment; Chemical, Biological, Radiological, Nuclear, and high yield Explosive (CBRNE) treatment; hypothermia treatment; and smoke inhalation treatment and monitoring.

Regarding the statements that attained consensus, the need for each first response agency to develop guidelines, education, and training on management of permissive hypotension, rapid vascular access, crush injury management, and pain relief were specifically related to treatment.

Prehospital processes refer to the organizational structures and operational management practices that coordinate deployment and utilization of resources, patient response, dispatch and transport, and non-clinical activities that organize prehospital capability to mass-casualty incident response. This study achieved consensus on 23 of the 28 statements that underwent modified Delphi review.

## Discussion

The expert consensus triage, prehospital life support and damage control, and prehospital process statements produced in this modified Delphi study inform the development of toolkits and clinical guidelines to respond to mass-casualty incidents to meet the NIGHTINGALE project objectives.

### Triage

Mass-casualty incident triage findings produced by this study are congruent with observed challenges or absence of validation of triage systems in mass-casualty incidents. Validation of triage practices has been predominantly informed by practice in daily care of traumatic patients rather than mass-casualty incidents. The challenge in doing so lies in the fact that the profile of daily practice circumstances is totally different. Delgado, et al have published findings of a triage system calculated from patients involved in a mass-casualty incident showing both sensitivity and specificity of tool accuracy.^19^

Consensus of the participants of this modified Delphi study proposed advancement of the current concept of mass-casualty incident triage, from the initial sorting of injured patients into a static triage category to a dynamic continuum of care. Achieving this would require continuous sorting of mass-casualty incident casualties as the resources of staff, stuff, and structure necessary to meet demand are deployed. The goal of mass-casualty incident triage is to identify those patients requiring life support and damage control interventions during all the phases of the priority transport to definitive care.

The importance of standardizing and employing a consistency in practice related to the continuum of mass-casualty incident triage is relevant when considering the existing literature, from which multiple mass-casualty incident triage practices developed globally have emerged. Bazyar, et al identified 20 different triage practices employed world-wide used for the initial assessment of mass-casualty incident victims with variations in sensitivity and sensitivity.^20^ Such variance creates a risk of the potential use of multiple or differing methods, which may result in suboptimal decision making and resource allocation.^20^ Compounding this, initial triage inaccuracy has been reported in research conducted by Kahn, et al who examined outcomes of Simple Triage and Rapid Treatment (START).^21^

In a mass-casualty incident with the mismatch between the demand of an unknown number of patients with unknown injuries and the dynamic accumulation of prehospital resources, the assignment of a triage category with re-assessments based on prehospital life support and damage control will change based on the treatment response and the volume of accumulating patients that are also undergoing treatment and re-assessments, which are competing for priority transport.

Priority transport decisions to various destinations in a mass-casualty incident are dynamic, as patients are not transported directly from the scene to the hospital. There are delays due to many factors with prehospital life support and damage control and other treatments to be administered in the field. The introduction of mass-casualty incident Key Performance Indicators, which examine the dynamic continuous sorting of patients to receive prehospital life support and damage control and priority transport, may enhance outcomes. Such practices have been considered; the findings of Gonzalos, et al resulted in the introduction of a “red surgical category,” which informs evacuation priority to the closest surgical hospital the respective patient may need.^22^

The outcomes of a systematic review conducted by Marcussen, et al showed inconsistency in initial mass-casualty incident triage allocation and accuracy between system types SIEVE,^23^ SMART Tag system,^24^ and CareFlight.^25^ The need for consistency in application is also supported by the accepted statement recommending triage education and training in this research. The application of resource-scarce mass-casualty incident triage is rarely a day-to-day skill of first responders; when required, staff should be conversant and skilled in its application to achieve the most significant outcome effect. Not having a robust education and training program risks inappropriate or inaccurate continuous triage, and as Kennedy, et al noted: “The disaster situation is not the time to try out a system for the first time.”^26^ This finding is consistent with the outcomes of this study recommending that triage systems should be simple, easy to remember, amenable to quick memory aids, and just-in-time training for trained ﬁrst responders. This encourages a jurisdiction to design or incorporate existing triage systems compatible with all first-responding agencies. The results of this modified Delphi study provide an opportunity to consider a nuanced approach to disaster triage that recognizes the context of population hazards and vulnerabilities. Further exploration of the efficacy in achieving and implementing this is warranted. Further insights on triage can be gathered by statements that did not achieve consensus. In particular, the modified Delphi participants did not reach consensus on statements related to monitoring. Given the focus of the NIGHTINGALE project technology developments, this finding is significant in the creation and development of any patient monitoring enhancements.

### Prehospital Life Support and Damage Control

Of the three areas of practice investigated, prehospital life support and damage control achieved limited consensus on proposed statements comparative to triage and processes (eight statements versus 18 and 23, respectively). Where consensus was achieved related to treatment, it was confined to specific areas of practice (ie, crush and hypotension). Crush injury guidance in disasters has most often been examined in post-earthquake settings, resulting in the development of consensus statements to provide guidance.^27,28^ The findings of this modified Delphi study underpin the need for responder education, training, and guidance in the management of crush injury as a possible consequence in all disaster types, as tornadoes, building collapses due to terrorism and asymmetric warfare, faulty construction, and other causes create a risk of crush injuries.

The consensus achieved on statements related to permissive hypotension and rapid vascular access using intraosseous access suggests that focused trauma management is specifically recommended by the modified Delphi experts. This is pertinent as the type, frequency, and impact of sudden-onset disasters are changing, placing risk in new areas that may not have been previously identified in hazard vulnerability analysis. Multiple scene mass-casualty incidents following a targeted terror attack or mass-shooting events of civilian populations and asymmetrical warfare create a need for improved capability of first responders and health care workers in trauma management. In a comprehensive review on permissive hypotension used to treat hemorrhagic shock following trauma, Albreiki, et al discovered that it is both practical and safe to use permissive hypotension to treat hemorrhagic shock in prehospital and in-hospital settings.^29^ The study by Albreiki, et al recommended further trials to assess the effectiveness of this practice on survival rates, in conjunction with the statement from this study that achieved consensus research in field use for disaster response, is warranted.^29^

The available research and evidence related to the provision of analgesia in disasters to inform practice is limited.

Whilst several conference proceedings and medical texts describing the current state of evidence in this area or proposing treatment practices exists, there is limited literature exploring pain relief practice after sudden-onset disasters.^30–36^ Key areas of analgesia practice in contemporary research focus on use of nerve block interventions and ketamine as an analgesic agent in the field.^37–40^ Stewart’s summary of potential options for consideration of field analgesia identifies need for further research and guidance on development of administration techniques suitable for field use and consideration of safe options of pain relief.^41^ This modified Delphi study recognizes and underpins the need for provision of analgesia in mass-casualty incident response and furthers the call for research to describe best practice and novel administration in resource-scarce mass-casualty incidents after sudden-onset disasters.

The need for obtaining patient consent was also identified by the modified Delphi experts. In the setting of a mass-casualty incident after a sudden-onset disaster, this is of particular significance in guidance to domestic and international response teams to inform an ethical response framework for care. Disaster ethics have been previously considered by Geale, et al as requiring further development and maturity that considers the scope of practice of responders and ensuring that the rights of the patient, including consent to treatment, are upheld in events resulting in mass-casualty incidents.^42^

### Prehospital Processes

Mass-casualty incident response prehospital processes are undergoing rapid change due to new and emerging technologies. Identification and adaptation of such technology adopted in other industries offers potential enhancement of existing prehospital response processes. Technological developments in telemedicine, artificial intelligence, drone technology, active shooter response, diagnostic equipment, and live data feeds of resource systems offer additional or enhanced tools to disaster responders. Such enhancements can potentially enable more effective priority transportation of patients with efficient and effective hospital distribution to match the patient with the most capable facility.^43^ Equally, the learning gained from active shooter mass-casualty incidents offers opportunities to update prehospital response plans and processes.^44^

The use of mobile apps to support triage and patient assessment was explored in a systematic review by Montano, et al who found that the development of apps should ensure accessibility and continuity of care between prehospital and hospital providers and include treatment guidelines for responders.^45^ Of note, the use of telemedicine to support mass-casualty incident response did not meet consensus as a proposed statement in this modified Delphi. It was also noted that proper testing should be conducted before field implementation when technology is to be introduced. Contemporary research shows that incidents of active armed offenders, particularly active shooter mass-casualty incidents, has consequences in some jurisdiction.^46^ Despite this, consensus was not achieved on statements related to active armed offender practice.

There was a difference in prehospital process statements achieving consensus regarding guidance for patient treatment by medical first responders versus first responders/first response agencies. This outcome may be related to the higher proportion of physicians participating in the modified Delphi proportionate to first responder participants. Defining futility of care did not reach consensus, mirroring the discussions across jurisdictions in many nations based on religious, cultural, and legal considerations. Mass-casualty incident crisis standards of care after sudden-onset disasters and allocation of scarce resources in such settings remains challenging for prehospital staff. The ethical application of such decisions has resulted in recommendations of establishing frameworks of practice a priori to sudden-onset disaster occurrence. However, the practical establishment of such, including clarity of decision making, remains needed in many situations.^42,47–50^

Whilst a Delphi review identifies where consensus is achieved, which subsequently informs guidance for practice, statements that did not achieve consensus may be equally informative. Statements that did not achieve consensus were thematically centered around clinical practices and patient care interventions. Given that a high proportion of Delphi participants were health care practitioners from across a diverse geographical area, a lack of consensus on care warrants further exploration such that common practices desired in care are articulated. The lack of prehospital life support and damage control consensus is a finding and suggests an urgent need for the undertaking of robust research to establish a more substantial evidence base to guide prehospital life support and damage control in the mass-casualty incident resource-scarce environment when the number of patients and their injuries is unknown.

## Strengths

Strengths of this project included the engagement of a diverse and international panel of participants of health care practitioners to participate in Delphi statement assessment and review.

## Limitations

This review is limited to study design and methodology. This modified Delphi study utilized pre-selected data informed by a PRISMA-ScR scoping review of prehospital triage, life support and damage control interventions, and the inherent processes to develop draft statements, in contrast to the more standard approach of an open questionnaire to collect such data from experts. The limitations of this process may not have captured relevant references to collect data to incorporate into the creation of the initial draft Delphi statements.

No standard minimum number of experts required for a Delphi study is known, however Franc, et al have described support for a minimum of five.^51^ Furthermore, experts are often selected as a function of their availability to perform the Delphi process, the scope of the consultation, or their expertise in that field.^14^ In addition, the selected number of experts needs to account for the fact that, from one round to another, the number of experts willing to participate can drop significantly.^52^ Therefore, specific authors recommend the number of experts to be lower than 50,^53^ while others consider larger values.^15^

In this modified Delphi study, there was an improved triage expert response and consistent prehospital life support and damage control expert response, with an inference that this had no bearing on the round-to-round consensus, with the final consensus attained based on stability of the statements not reaching consensus after three rounds. There is no way to know of the varied prehospital process expert response impacted round-to-round consensus with the final consensus attained based on stability of the statements not reaching consensus after three rounds.

Consequently, several methodological decisions were required, including the number of Delphi rounds undertaken, the threshold for defining consensus, and the selection of the experts for Delphi round participation. A common practice for Delphi studies is to cease further rounds when consensus is reached.^52^ The optimal number of rounds and the acceptable level of consensus can vary depending on the number of expert participants and the a priori target of the Delphi process undertaken.^52,54^

## Conclusion

This project provided guidance to enhance mass-casualty incident response triage, prehospital damage and life support, and prehospital processes through the modified Delphi scientific process. The consensus statements, in addition to the data collected in the scoping review, will be utilized to inform the creation of mass-casualty incident response toolkits and clinical guidelines in the NIGHTINGALE project.
